# Injury prevention in Super-G alpine ski racing through course design

**DOI:** 10.1038/s41598-021-83133-z

**Published:** 2021-02-11

**Authors:** Matthias Gilgien, Philip Crivelli, Josef Kröll, Live S. Luteberget, Erich Müller, Jörg Spörri

**Affiliations:** 1 Department of Physical Performance, Norwegian School of Sport Sciences, Oslo, Norway; 2 Center of Alpine Sports Biomechanics, Engadin Health and Innovation Foundation, Samedan, Switzerland; 3 WSL-Institute for Snow and Avalanche Research SLF, Group for Snowsports, Davos, Switzerland; 4 Department of Sport Science and Kinesiology, University of Salzburg, Hallein-Rif, Austria; 5 Sports Medical Research Group, Department of Orthopaedics, Balgrist University Hospital, University of Zurich, Zurich, Switzerland; 6 University Centre for Prevention and Sports Medicine, Department of Orthopaedics, Balgrist University Hospital, University of Zurich, Zurich, Switzerland

**Keywords:** Risk factors, Engineering, Mathematics and computing, Physics

## Abstract

In Super-G alpine ski racing mean speed is nearly as high as in Downhill. Hence, the energy dissipated in typical impact accidents is similar. However, unlike Downhill, on Super-G courses no training runs are performed. Accordingly, speed control through course design is a challenging but important task to ensure safety in Super-G. In four male World Cup alpine Super-G races, terrain shape, course setting and the mechanics of a high-level athlete skiing the course were measured with differential global navigation satellite systems (dGNSS). The effects of course setting on skier mechanics were analysed using a linear mixed effects model. To reduce speed by 0.5 m/s throughout a turn, the gate offset needs to be increased by + 51%. This change simultaneously leads to a decrease in minimal turn radius (− 19%), an increase in impulse (+ 27%) and an increase in maximal ground reaction force (+ 6%). In contrast, the same reduction in speed can also be achieved by a − 13% change in *vertical gate distance*, which also leads to a small reduction in minimal turn radius (− 4%) impulse (− 2%), and no change in maximal ground reaction force; i.e. fewer adverse side effects in terms of safety. It appears that shortening the vertical gate distance is a better and safer way to reduce speed in Super-G than increasing the gate offset.

## Introduction

In each World Cup (WC) alpine ski racing competition season, approximately one third of all athletes suffer an injury^1–3^. Including overuse injuries and injuries sustained off season, the rates are even higher^4,5^. Accordingly, alpine ski racing is recognized as having one of the highest injury risks of all Olympic winter sports^6–8^.

In Super-G and Downhill speed is high compared to the other disciplines^9,10^. Since speed is the driving factor for forces that are dissipated during crashes and collisions with the surroundings^11^, impact injuries are frequent in these two competition disciplines^12^. Consequently, spill zones and impact protective equipment such as netting along the race courses are commonly used countermeasures^13^. Additionally a reduction of speed in general, and before potential injury hotspots in particular, may be helpful in preventing impact injuries^14^. An earlier study of Downhill races assessed whether speed, as a key driver for impact injuries, could be reduced by changes in ski geometry and binding plates. The study, however, revealed that equipment alterations have only small effects on speed and that lower speed does not substantially increase the time athletes have to anticipate and prepare for technically difficult situations in Downhill^15^. Similar changes in equipment did not have substantial effects on speed in Giant Slalom (GS), but did positively affect the skiers' motion patterns and kinetics^16,17^. Hence, studies suggest it is difficult to adequately increase ski–snow friction forces via changes in equipment properties, particularly in the speed disciplines of Super-G and Downhill.

An alternative approach to reducing speed in Super-G or Downhill is course design. Recent studies have shown that terrain incline^18^, turn entrance speed^18^ and course setting^18–21^ are measures that have an effect on skier speed in technical disciplines. For GS it was found that increasing the gate offset or reducing the vertical gate distance both reduced speed^18^. However, increasing the gate offset to reduce speed caused an increase in injury risk through increased impulse (integration of the external forces over time as a measure of the athletes physical effort, the effort that will lead to fatigue throughout the race), reduced minimal turn radius and increased maximal force, while a reduction in the vertical gate distance did not lead to such increases in injury risk^18^. It is, however, unknown whether speed can be controlled in Super-G in a similar manner to that demonstrated for GS, and it would be beneficial if similar quantitative recommendations could be generated for Super-G. It would also be helpful to determine the negative side effects of course-setting manipulations on other injury risk factors, such as turn radius, impulse and ground reaction forces. Therefore, this study aimed to: (1) assess whether course-setting characteristics, entrance speed and terrain incline influence speed through a turn; and (2) assess whether changes in course-setting aimed at reducing speed have negative consequences on turn radius, impulse and turn forces in Super-G.

## Methods

### Study protocol

During two winter seasons, data was captured at four male Super-G World Cup races in Kitzbühel (AUT), Hinterstoder (AUT), and twice in Crans Montana (SUI). The races were chosen to cover the range of typical World Cup Super-G courses. A forerunner, testing the course directly prior to competition, was tracked using a differential high-end global navigation satellite carried by the skier. For each location a different forerunner was chosen to ski the course. The forerunners were athletes competing at European Cup level. The study was approved by the Ethics Committee of the Department of Sport Science and Kinesiology at the University of Salzburg. Each subject provided written informed consent prior to taking part in the study and the methods were carried out in accordance with the relevant guidelines and regulations.

### Data collection

Start, turning gate positions, finish line and the terrain were captured prior to the respective race using static dGNSS: Alpha-G3T receivers with GrAnt-G3T antenna (Javad, USA) and Leica TPS 1230 + (Leica Geosystems AG, Switzerland). To reconstruct the snow surface in enough detail, on average 0.3 points per m^2^ were measured. At terrain transitions this number was substantially increased and was reduced in uniform terrain^9,10^.

Athlete tracking during skiing was conducted using a high-end differential global navigation satellite system (dGNSS) carried by the athlete on the back and helmet^22^. A GPS/GLONASS dual frequency (L1/L2) receiver (Alpha-G3T, Javad, USA) collected antenna position at 50 Hz. The antenna (G5Ant-2AT1, Antcom, USA) was mounted on the forerunner’s head. To calculate the carrier phase double difference position solutions, two (for redundancy) base stations were located at the start of the course and equipped with GNSS antennas (GrAnt-G3T, Javad, USA) and Alpha-G3T receivers (Javad, USA).

### Geodetic reconstruction of skier trajectory and snow surface

Static dGNSS measurements on the terrain surface to represent the snow surface, and gate positions to represent the course, were calculated in a post-processing procedure, using the geodetic GNSS software Justin (Javad, San Jose, USA)^9,10^. To calculate skier antenna position data, double difference carrier phase solutions were processed from GPS and GLONASS satellites and frequency L1/L2 using the geodetic software’s (GrafNav NovAtel Inc., Canada) KAR algorithm^22^. Turns for which the geodetic solution ambiguities failed to be solved were excluded from the study.

### Calculation of course characteristics

The shape of the snow surface was surveyed using static dGNSS. A digital terrain model was generated from the point cloud captured by dGNSS by triangulation of the point cloud, using the Delaunay method, and gridded on a rectangular grid^9,10^. The local incline of the terrain was geometrically derived using the local terrain surface normal vectors. To represent the average slope incline of a turn, the local incline vectors associated with a turn were averaged over the area of each turn^9,10^. The average turn incline was named *Terrain*_*INCLINE*_ and expressed as the angle to the horizontal^9,10^.

**Figure 1:**
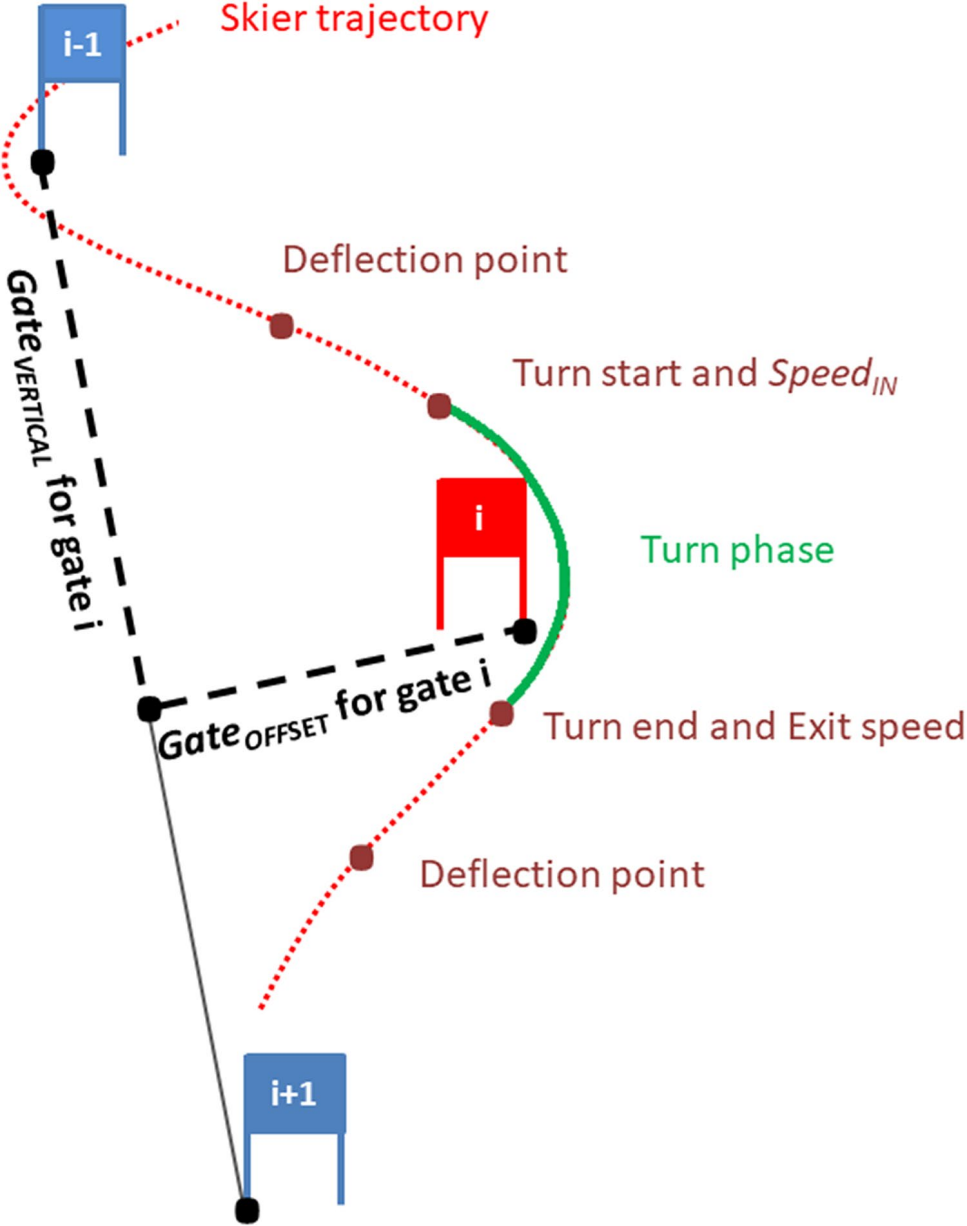
Definition of the course setting characteristics in black: vertical gate distance (*Gate*_*VERTICAL*_) and gate offset (*Gate*_*OFFSET*_). The turn phase is colored in green. In red: turn phase starts within two deflection points and starts where the center of mass turn radius drops below 75 m and ends where the center of mass turn radius exceeds 75 m. Speed at turn start (*Speed*_*IN*_) and turn exit speed are measured at turn start and turn end.

Courses in alpine skiing are marked with gates, with a turning gate and a gate on the outside of the line skiers take. The setting of the course is characterized by two gate distances; the vertical gate distance (*Gate*_*VERTICAL*_) and the gate offset (*Gate*_*OFFSET*_), also named horizontal gate distance in other studies (see Fig. 1)^9,10,20^. The Euclidian distance between turning gates, also called the linear gate distance, was not added to the analysis, since the linear gate distance is linearly highly dependent on *Gate*_*VERTICAL*_, and independent of *Gate*_*OFFSET*_. *Gate*_*VERTICAL*_ was the distance from gate (i − 1) to the projection of gate (i) onto the vector between (i − 1) and (i + 1), as shown in Gilgien et al.^9,10^. *Gate*_*OFFSET*_ for gate (i) was calculated as the normal projection of gate (i) on the vector from gate (I − 1) to gate (i + 1). When two consecutive gates formed a delay turn (a long turn marked with two gates) the gate with the larger *Gate*_*OFFSET*_ was selected as the one to represent the turn^9,10^.

### Calculation of the skier mechanics

The position of the dGNSS antenna that was mounted on the helmet of the athlete was filtered using bi-cubic spline functions. To better estimate the center of mass (CoM) position, a mechanical pendulum model was attached to the antenna position with a global position accuracy of 9 ± 12 cm^23^. Instantaneous speed, turn radius, and ground reaction force and impulse were time-derived from the pendulum center of mass position data^24^. The start and end of a turn were defined as the phase between two deflection points of the skier’s CoM trajectory (projected in the horizontal plane) when the CoM turn radius was below 75 m^14^. The speed at which an athlete entered a turn (*Speed*_*IN*_) was defined as the instantaneous speed at the point when the turn radius dropped below 75 m prior to the gate passage. Exit speed was the instantaneous speed at the point when the turn radius exceeded 75 m for the first time after the gate passage. The change in speed through a turn (*∆Speed*) was computed as the difference of *Speed*_*IN*_ and Exit speed. The minimal turn radius through a turn (*Radius*_*MIN*_) was also considered as an injury proxy and was defined as the smallest radius in the turn^14,18,20^. The maximal ground reaction force (*GRF*_*MAX*_) was defined as the highest instantaneous ground reaction force during a turn^24^. To measure the effort athletes excerpt to the surrounding over the time of the course impulse was calculated as the time integration of the external forces. Impulse might also serve as an approximation of accumulated fatigue since both duration and the intensity of the physical effort are reflected in this measure. Impulse as a proxy of fatigue was defined as the integral of ground reaction force and air drag (in body weight) through the turn time and expressed in body weight seconds (BWs)^14,25^.

### Statistical analysis

The data from all four races were pooled together to create a dataset of 192 turns. To characterize the central tendency and variability of the course setting and athlete mechanical parameters, mean and standard deviation (SD) were calculated.

To determine the relationship between course setting and adjustments in course setting and their effect on skier mechanics, a linear mixed effects model was established (192 turns; MIXED procedure in SAS 9.4 Software, Raleigh, USA)). Since snow conditions were not quantified and the forerunners were different in each race location, the model contained the random variables “Skier ID” (3 different athletes), and “Skier ID*Race ID”. As model predictors we included the variables: *Speed*_*IN*_, *Terrain*_*INCLINE*_, *Gate*_*OFFSET*_ and *Gate*_*VERTICAL*_. The mixed model was run for each outcome variable: *Radius*_*MIN*_, *∆Speed*, *GRF*_*MAX*_ and Impulse. To generate comparable results between the different outcome variables, the fixed effects of the model were adjusted by division by two SD. The results of this intermediate step calculation can be found in the Supplementary material. With this scaling of the results, the model can be interpreted in the sense of “How much change in *Gate*_*VERTICAL*_ is required to change *∆Speed* by two standard deviations?".

The mixed model was re-run on all turns to calculate the adjustment required for each predictor variable (*Speed*_*IN*_, *Terrain*_*INCLINE*_, *Gate*_*OFFSET*_ and *Gate*_*VERTICAL*_) to result in a reduction in speed of 0.5 m/s (*∆Speed* = − 0.5 m/s) from turn start to turn end. The effect that this change in the predictors (to reduce speed by 0.5 m/s) had on the other outcome variables, *Radius*_*MIN*_, *GRF*_*MAX*_ and Impulse, was also determined.

## Results

Table 1 provides an overview of the central tendency, variability and range (mean, SD, minimal and maximal values) of the predictor and outcome variables of the mixed model and reveals that, in the competition discipline Super-G, *Gate*_*OFFSET*_ has much higher variability than *Gate*_*VERTICAL*_ or linear gate distance.

**Table 1:** Mean, SD, and minimal and maximal values for predictors and outcome variables of the mixed model for Super-G.

	Mean	SD	Min	Max
Linear gate distance	50.27	8.54	32.03	147.91
*Gate*_*OFFSET*_ [m]	13.32	7.45	0.16	38.56
*Gate*_*VERTICAL*_ [m]	47.80	9.09	28.99	145.45
*Speed*_*IN*_ [m/s]	24.16	2.55	16.33	30.66
*Terrain*_*INCLINE*_ [°]	19.01	5.48	5.44	34.85
*∆Speed* [m/s]	0.15	1.88	− 5.15	7.17
*Radius*_*MIN*_ [m]	35.17	15.66	9.94	128.33
*GRF*_*MAX*_ [BW]	2.38	0.57	1.03	4.43
Impulse [BWs]	3.24	1.07	0.61	7.29

### Effects of adjustments in course setting, entrance speed and terrain incline on speed reduction

Table 2 illustrates the results of the linear mixed effects model describing the effect that changes in course setting, terrain incline, and *Speed*_*IN*_ have on *∆Speed* (reduction in speed induced by the given adjustments through a turn). All model predictors have a significant effect on speed.

**Table 2:** Results showing what change in each predictor is needed to cause a reduction in speed (*∆Speed*) of − 0.5 m/.

*∆Speed* [m/s]	p value	Absolute change in m required to reduce speed by 0.5 m/s	Relative change required in % to reduce speed by 0.5 m/s
Predictor *Gate*_*OFFSET*_ [m]	< 0.0001	6.84	51
Predictor *Gate*_*VERTICAL*_ [m]	< 0.0001	− 6.31	− 13
Predictor *Speed*_*IN*_ [m/s]	< 0.0001	2.2	9
Predictor *Terrain*_*INCLINE*_ [°]	< 0.0001	− 3.68	− 19

The underlying analysis for these results is provided in the supplementary material in Tables 4 and 5.

To achieve a reduction in speed of 0.5 m/s throughout a turn, *Gate*_*OFFSET*_ needs to be increased by 51% of the existing *Gate*_*OFFSET*_, or an average of 6.84 m. For the same decrease, *Gate*_*VERTICAL*_ would need to be shortened by − 6.31 m, which is − 13% of the existing *Gate*_*VERTICAL*._. Further, if course setting and terrain is held constant, but skiers enter the turn at 2.2 m/s or 9% higher entrance speed (*Speed*_*IN*_), due to the phenomenon of the "velocity barrier" (i.e. a skier’s voluntary control of speed in order to avoid mistakes), they would lose 0.5 m/s speed throughout the turn. And, for a given course setting and *Speed*_*IN*_, a 3.68° steeper *Terrain*_*INCLINE*_ would result in a speed reduction through the turn of 0.5 m/s compared to a turn with the given *Terrain*_*INCLINE*_.

### Effects of adjustments in course setting on turn radius, ground reaction force and impulse

The data in Table 3 shows that a − 0.5 m/s reduction in speed through a turn *(∆Speed)* caused by an increase in *Gate*_*OFFSET*_ by + 6.84 m (Table 2) typically leads to a − 6.57 m smaller *Radius*_*MIN*_, which corresponds to a reduction of − 19%. In contrast, a − 0.5 m/s speed reduction through a turn caused by a − 6.31 m shorter *Gate*_*VERTICAL*_ (Table 2) leads to a substantially smaller reduction in *Radius*_*MIN*_ (absolute: − 1.40 m / percentage: − 4%).

**Table 3:** Results showing what effect reductions in speed (*∆Speed*) of − 0.5 m/s through course setting adjustments have on *Radius*_*MIN*_, *GRF*_*MAX*_ and impulse.

*Radius*_*MIN*_ [m]	p value	Absolute reduction in *Radius*_*MIN*_ in m as a consequence of speed reduction of 0.5 m/s	Relative reduction in *Radius*_*MIN*_ in % as a consequence of speed reduction of 0.5 m/s
Predictor *Gate*_*OFFSET*_ [m]	< 0.0001	− 6.57	− 19
Predictor *Gate*_*VERTICAL*_ [m]	0.0100	− 1.40	− 4

*GRF*_*MAX*_ [BW]	p value	Absolute reduction in *GRF*_*MAX*_ in BW as a consequence of speed reduction of 0.5 m/s	Relative reduction in *GRF*_*MAX*_ in % as a consequence of speed reduction of 0.5 m/s
Predictor *Gate*_*OFFSET*_ [m]	< 0.0001	0.13	6
Predictor *Gate*_*VERTICAL*_ [m]	0.6700	–	

Impulse [BWs]	p value	Absolute reduction in impulse in BWs as a consequence of speed reduction of 0.5 m/s	Relative reduction in impulse in % as a consequence of speed reduction of 0.5 m/s
Predictor *Gate*_*OFFSET*_ [m]	< 0.0001	0.87	27
Predictor *Gate*_*VERTICAL*_ [m]	0.0100	− 0.07	− 2

The results must be read as follows: e.g. an increase of *Gate*_*OFFSET*_ of 6.84 m (Table 2), leading to a speed reduction (*∆Speed*) of − 0.5 m/s causes a reduction in *Radius*_*MIN*_ of − 6.57 m. The results for Predictor *Speed*_*IN*_ and Predictor *Terrain*_*INCLINE*_ are provided in the supplementary material in Table 6.

Moreover, an increase in *Gate*_*OFFSET*_ of + 6.84 m (Table 2) to reduce speed by − 0.5 m/s leads to a small but significant increase in *GRF*_*MAX*_ of + 0.13 BW (i.e. + 6%). No increase in *GRF*_*MAX*_ is found if *Gate*_*VERTICAL*_ is shortened by − 6.31 m to reduce speed by − 0.5 m/s.

Finally, impulse increases by + 0.87 BWs (i.e. + 27%) if *Gate*_*OFFSET*_ is increased by + 6.84 m to reduce speed by − 0.5 m/s. In contrast, impulse is actually decreased by − 0.07 BWs (i.e. − 2%) if *Gate*_*VERTICAL*_ is shortened by − 6.31 m to reduce speed by − 0.5 m/s.

## Discussion

The main findings of this study were: increasing *Gate*_*OFFSET*_ to reduce speed by − 0.5 m/s throughout a turn (+ 6.84 m; + 51%) simultaneously leads to a decrease in *Radius*_*MIN*_ (− 19%), an increase in Impulse (+ 27%) and an increase in *GRF*_*MAX*_ (+ 6%). To reduce speed by − 0.5 m/s with a change in *Gate*_*VERTICAL*_*,* compared to *Gate*_*OFFSET*_, a much smaller percentage change is required (− 6.31; − 13%). Further, shortening *Gate*_*VERTICAL*_ to reduce speed by − 0.5 m/s causes a much smaller reduction in *Radius*_*MIN*_ (− 4%) and Impulse (− 2%), and no increase in *GRF*_*MAX*_.

As found in this study, course-setting characteristics, entrance speed, and terrain incline are key contributors to speed in Super-G alpine ski racing, in which course setting is directly modifiable for preventative purposes. Both increasing *Gate*_*OFFSET*_ and decreasing *Gate*_*VERTICAL*_ lead to a reduction of speed; however, for the same speed reduction (− 0.5 m/s) the required percentage changes are almost four times higher for *Gate*_*OFFSET*_ (+ 51%) than for *Gate*_*VERTICAl*_ (− 13%), while the absolute changes are similar (Table 2). An earlier study revealed this percentage-wise difference to be also present in GS; however, in GS the magnitudes only differed by a factor of two, not four as in the current study (see Table 7 in supplementary material). Hence, a relatively smaller increase in *Gate*_*OFFSET*_ was required to reduce speed compared to shortening of *Gate*_*VERTICAl*_^18^. This substantial difference between Super-G and GS may be related to ski–snow interaction. In Super-G, athletes are likely able carve the turn to larger extent and use less pivoting or skidding than in GS. This is supported by the finding that skidding in downhill and slalom causes a massive increase in ski-snow friction compared to carving^15,21,26^.

Similar to the findings of earlier studies in slalom and giant-slalom, higher entrance speed led to a higher speed reduction (energy dissipation) throughout the turn. This finding further supports the presence of a so called "velocity barrier"; the idea that skiers need to dissipate energy (control speed by braking) at certain points along the course in order to avoid making mistakes^16,18,27^. The current study, however, described this phenomenon for the first time for Super-G alpine skiing. Finally, terrain inclination also plays an important role in speed control in Super-G. This study showed that a reduction of − 19% (3.68°) in terrain incline reduced speed by 0.5 m/s, which is in line with previous observations in other disciplines^18,28^. However, as entrance speed and terrain are only indirectly modifiable by course setting for a specific race venue, they must be considered as important confounders rather than manipulatable prevention approaches.

The results of this study show that course setters can control and reduce speed by either increasing *Gate*_*OFFSET*_ or decreasing *Gate*_*VERTICAL*_. However, the different percentage-wise impact on speed-reduction (larger for decreasing *Gate*_*VERTICAL*_) is not the only relevant aspect in the decision of whether to control speed by adjustments in *Gate*_*OFFSET*_ or *Gate*_*VERTICAL*_*.* As shown in this study, the increase in *Gate*_*OFFSET*_ to reduce speed by 0.5 m/s resulted in an adverse decrease in minimal turn radius, an increase in maximal ground reaction forces and an increase in impulse (Table 3). As these factors are well-known drawbacks causing course setting modifications to adversely affect the load exposure, balance and fatigue of the skiers^14,20,29^, the conclusion that shortening *Gate*_*VERTICAL*_ is to be favored over increasing *Gate*_*OFFSET*_*,* as previously found for GS, is even more evident in Super-G^18^ (see Table 7 in supplementary material). Moreover, similar to GS^16,18^, in the speed disciplines SG and Downhill, the potential effects of reducing speed by adjusting course setting (current study) appear much higher than those achieved with modified ski equipment^15^. To date, it has not been scientifically investigated whether a clothing-induced increase in air drag could adequately reduce speed in the speed disciplines^25,30,31^. Additional padding and use of race suit fabrics with higher air drag coefficient would increase air drag and are likely to reduce speed. Padding might also improve crash impact absorption. Snow properties may also have substantial impact on speed^32^, but these are only partly in the race organizers’ hands, especially in SG and downhill, where courses are long and environmental factors play an important role in the generation of snow properties.

Preventing injuries in Super-G is challenging, since (1) mean speed is only slightly lower (− 2 m/s) than in Downhill^9,10^ and consequently the impact energy in impact accidents is only slightly lower than in Downhill; (2) courses are technically more challenging; and (3) skiers do not have training sessions on the courses prior to competitions as is the case in Downhill. More specifically, Super-G courses are steeper, include more turns with smaller radii and more frequent and more pronounced terrain transitions^9,10^. Ground reaction forces are higher and the total physical load (impulse) of a competition is only 13% lower than in downhill^25^. Super-G courses also include an average of 2.3 jumps (4.2 for downhill), with jump length being only 21% and airtime being only 6% shorter than in Downhill^14^. Hence, jumps in Super-G can be as challenging as in Downhill, since a given rotation impulse at take-off can cause nearly the same off-balance rotation as in Downhill^14^.

In Downhill it is a safety requirement that athletes need to participate in at least one official training run on the course prior to competition to become familiar with the course. In Super-G athletes only have the opportunity to inspect the course some hours prior to competition and need to anticipate their skiing strategy and find a balance between performance and safety. Preventing injuries in Super-G is not only difficult for skiers, but also for organizers and course setters. In Downhill, course setting is kept similar from year to year, which allows organizers to learn and improve courses over the years, while Super-G courses are set differently by different coaches each year and change accordingly. Hence, course setters and the race directors of the International Ski Federation FIS, who carry the ultimate responsibility for course safety, have no opportunity to test the Super-G course for safety prior to competition. Therefore, experience in course setting is important. However, this might take years to build, since WC coaches only set about 40 Super-G training courses per year^33^; mostly on easier and shorter slopes than in competition. In this connection, the findings of the current study may help course setters to develop a better understanding of how to set safe courses and adequately control speed in Super-G.

There are some limitations to be aware of when interpreting the study findings:

First, the number of turns was not sufficient to split the analysis into terrain incline sub-groups as shown in a similar study on GS^18^. This leaves us with the problem that we cannot provide information on how adjustments in course setting depend on terrain incline. Second, the study analysis did not look at the interaction of consecutive turns or entire course sequences and hence, experimental course set testing for a given situation seems indicated. Third, the applied dGNSS methodology did not provide direct data describing the influence of course setting on the mode of ski–snow interaction. Thus, we can only speculate as to why speed is reduced as a function of ski–snow interaction. Future studies should investigate the effect of course setting on skidding and the underlying ski–snow interaction in more detail. For the four races, three different athletes were employed as forerunners. The effect of forerunner however was compensated for in the mixed model.

## Supplementary Information

Supplementary Information.
