# Supplementary material for: Injury prevention in Super-G alpine ski racing through course design
Source: Sci Rep. 2021 Feb 11;11:3637. doi: 10.1038/s41598-021-83133-z (PMC7878813; doi:10.1038/s41598-021-83133-z)
Supplement: Supplementary file 1 — Supplementary Information. [file 41598_2021_83133_MOESM1_ESM.docx]

# SUPPLEMENTARY MATERIAL

Injury prevention in Super – G alpine ski racing through course design

Matthias Gilgien^1,2^ , Philip Crivelli^3^, Josef Kröll ^4^, Live S. Luteberget ^1^, Erich Müller ^4^, Jörg Spörri ^5,6^

^1^ Department of Physical Performance, Norwegian School of Sport Sciences, Oslo, Norway

^2^ Center of Alpine Sports Biomechanics, Engadin Health and Innovation Foundation, Samedan, Switzerland

^3^ WSL—Institute for Snow and Avalanche Research SLF, Group for Snowsports, Davos, Switzerland

^4^ Department of Sport Science and Kinesiology, University of Salzburg, Hallein-Rif, Austria

5 Sports Medical Research Group, Department of Orthopaedics, Balgrist University Hospital, University of Zurich, Switzerland

^6^ University Centre for Prevention and Sports Medicine, Balgrist University Hospital, University of Zurich, Switzerland

***Correspondence to [Matthias.gilgien@nih.no]**

**Table 4:** Two standard deviations used to express the effects in the mixed model (Table 6).

|  | **2SD** |
| --- | --- |
| **Linear Gate Distance [m]** | 17.07 |
| ***Gate_OFFSET_* [m]** | 14.91 |
| **Gate_VERTICAL_ [m]** | 18.18 |
| ***Terrain_INCLINE_* [°]** | 10.96 |
| ***Speed_IN_* [m/s]** | 5.10 |

**Table 5:** The results of the mixed model expressed as a function of 2 standard deviations from Table 6. The table must be read as follows for the example of change in *Gate_OFFSET_* on *∆Speed*. A change of 2 SD in *Gate_OFFSET_* (14.91m from table 5) leads to a reduction in speed (*∆Speed*) of -1.09m/s.

| ***∆Speed* [m/s]** | **Estimate** | **p value** | **Lower Limit** | **Upper limit** |
| --- | --- | --- | --- | --- |
| **Intercept** | 0.15 | 0.21 | -0.16 | 0.46 |
| ***Gate_OFFSET_* [m]** | -1.09 | <.0001 | -1.48 | -0.70 |
| ***Gate_VERTICAL_* [m]** | 1.44 | <.0001 | 1.05 | 1.84 |
| ***Speed_IN_* [m/s]** | -1.16 | <.0001 | -1.57 | -0.75 |
| ***Terrain_INCLINE_* [°]** | 1.49 | <.0001 | 1.07 | 1.91 |
|  |  |  |  |  |
| **Radius_MIN_ [m]** | **Estimate** | **p value** | **Lower Limit** | **Upper limit** |
| **Intercept** | 36.80 | 0.00 | 22.18 | 51.42 |
| ***Gate_OFFSET_* [m]** | -14.32 | <.0001 | -17.33 | -11.30 |
| ***Gate_VERTICAL_* [m]** | 4.04 | 0.01 | 0.94 | 7.13 |
| ***Speed_IN_* [m/s]** | 8.58 | <.0001 | 5.12 | 12.03 |
| ***Terrain_INCLINE_* [°]** | -0.13 | 0.94 | -3.48 | 3.22 |
|  |  |  |  |  |
| **GRF_MAX_ [BW]** | **Estimate** | **p value** | **Lower Limit** | **Upper limit** |
| **Intercept** | 2.32 | 0.00 | 1.64 | 3.00 |
| ***Gate_OFFSET_* [m]** | 0.29 | <.0001 | 0.16 | 0.41 |
| ***Gate_VERTICAL_* [m]** | -0.03 | 0.67 | -0.16 | 0.10 |
| ***Speed_IN_* [m/s]** | 0.30 | <.0001 | 0.15 | 0.44 |
| ***Terrain_INCLINE_* [°]** | 0.07 | 0.34 | -0.07 | 0.21 |
|  |  |  |  |  |
| **Impulse [BWs]** | **Estimate** | **p value** | **Lower Limit** | **Upper limit** |
| **Intercept** | 3.24 | <.0001 | 3.12 | 3.36 |
| ***Gate_OFFSET_* [m]** | 1.89 | <.0001 | 1.73 | 2.04 |
| ***Gate_VERTICAL_* [m]** | 0.20 | 0.01 | 0.05 | 0.35 |
| ***Speed_IN_* [m/s]** | -0.01 | 0.92 | -0.17 | 0.15 |
| ***Terrain_INCLINE_* [°]** | 0.03 | 0.68 | -0.13 | 0.20 |

**Table 6:** Results showing the effect of a reduction in speed (*∆Speed*) of -0.5 m/s on *Radius_MIN_*, *GRF_MAX_* and on the predictors *Gate_OFFSET_* and *Gate_VERTICAL_*_,_ *Speed_IN_*, *Terrain_INCLINE_*. This is the same Table as Table 3 in the manuscript, but it includes all predictors from the mixed model. The results must be read as follows for the effect on *Radius_MIN_* through a change in *Gate_OFFSET_*: An increase of *Gate_OFFSET_* of 6.84m (Table 7) leads to a speed reduction (*∆Speed*) of -0.5m/s and reduces *Radius_MIN_* by -6.57m.

| **Radius_MIN_ [m]** | **p value** | **Absolute Reduction in Radius_MIN_ in m as a consequence of speed reduction of 0.5m/s** | **Relative Reduction in Radius_MIN_ in % as a consequence of speed reduction of 0.5m/s** |
| --- | --- | --- | --- |
| **Predictor *Gate_OFFSET_* [m]** | <.0001 | -6.57 | -19 |
| **Predictor *Gate_VERTICAL_* [m]** | 0.0100 | -1.40 | -4 |
| **Predictor *Speed_IN_* [m/s]** | <.0001 | 3.70 | 11 |
| **Predictor *Terrain_INCLINe_* [°]** | 0.9400 |  |  |
|  |  |  |  |
|  |  |  |  |
| **GRF_MAX_ [BW]** | **p value** | **Absolute Reduction in GRF_MAX_ in m as a consequence of speed reduction of 0.5m/s** | **Relative Reduction in GRF_MAX_ in % as a consequence of speed reduction of 0.5m/s** |
| **Predictor *Gate_OFFSET_* [m]** | <.0001 | 0.13 | 6 |
| **Predictor *Gate_VERTICAL_* [m]** | 0.6700 |  |  |
| **Predictor *Speed_IN_* [m/s]** | <.0001 | 0.13 | 5 |
| **Predictor *Terrain_INCLINE_* [°]** | 0.3400 |  |  |
|  |  |  |  |
|  |  |  |  |
| **Impulse [BWs]** | **p value** | **Absolute Reduction in Impulse in m as a consequence of speed reduction of 0.5m/s** | **Relative Reduction in Impulse in % as a consequence of speed reduction of 0.5m/s** |
| **Predictor *Gate_OFFSET_* [m]** | <.0001 | 0.87 | 27 |
| **Predictor *Gate_VERTICAL_* [m]** | 0.0100 | -0.07 | -2 |
| **Predictor *Speed_IN_* [m/s]** | 0.9200 |  |  |
| **Predictor *Terrain_INCLINE_* [°]** | 0.6800 |  |  |

* "Reduction in *Radius_MIN_* " refers to the Reduction in Radius_MIN_ as a consequence of a speed reduction of 0.5m/s

** "Increase in *GRF_MAX_* " refers to the Increase in GRF_MAX_ as a consequence of a speed reduction of 0.5m/s

*** "Increase in Impulse " refers to the Increase in Impulse as a consequence of a speed reduction of 0.5m/s

**Table 7:** Comparison of the data for Super – G given also in the manuscript in Table 2 and 3 with the data from the GS study. ^18^ For GS all turns were pooled into one group with 571 turns and analysed with the same mixed model approach as in this study on Super – G and the GS study. ^18^ The right-hand section of the table shows the absolute and relative differences between Super–G and GS.

|  | **Super - G** | | |  | **Giant Slalom** | | |  | **Difference between SG and GS (SG - GS)** | |
| --- | --- | --- | --- | --- | --- | --- | --- | --- | --- | --- |
|  |  |  |  |  |  |  |  |  |  |  |
| *∆Speed* [m/s] | **p value** | **Change required to reduce speed by 0.5 m/s** | **Change required in % to reduce speed by 0.5 m/s** |  | **p value** | **Change required to reduce speed by 0.5 m/s** | **Change required in % to reduce speed by 0.5 m/s** |  | **Change required to reduce speed by 0.5 m/s** | **Change required in % to reduce speed by 0.5 m/s** |
| Predictor *Gate_OFFSET_* [m] | <.0001 | 6.84 | 51 |  | <.0001 | 2.55 | 36 |  | 4.29 | 16 |
| Predictor *Gate_VERTICAL_* [m] | <.0001 | -6.31 | 13 |  | 0.00 | -7.60 | 30 |  | 1.29 | -17 |
| Predictor *Speed_IN_* [m/s] | <.0001 | 2.2 | 9 |  | <.0001 | 2.75 | 15 |  | -0.55 | -6 |
| Predictor *Terrain_INCLINE_* [°] | <.0001 | -3.68 | 19 |  | <.0001 | -6.49 | 32 |  | 2.81 | -13 |
|  |  |  |  |  |  |  |  |  |  |  |
| **Radius_MIN_ [m]** | **p value** | **Reduction in Radius_MIN_ in m as a consequence of speed reduction of 0.5m/s** | **Reduction in Radius_MIN_ in % as a consequence of speed reduction of 0.5m/s** |  | **p value** | **Reduction in Radius_MIN_ in m as a consequence of speed reduction of 0.5m/s** | **Reduction in Radius_MIN_ in % as a consequence of speed reduction of 0.5m/s** |  | **Reduction in Radius_MIN_ in m as a consequence of speed reduction of 0.5m/s** | **Reduction in Radius_MIN_ in % as a consequence of speed reduction of 0.5m/s** |
| Predictor *Gate_OFFSET_* [m] | <.0001 | -6.57 | 19 |  | <.0001 | -3.39 | 47 |  | -3.18 | -29 |
| Predictor *Gate_VERTICAL_* [m] | 0.01 | -1.4 | 4 |  | 0.42 |  |  |  |  |  |
|  |  |  |  |  |  |  |  |  |  |  |
| **GRF_MAX_ [BW]** | **p value** | **Reduction in GRF_MAX_ in m as a consequence of speed reduction of 0.5m/s** | **Reduction in GRF_MAX_ in % as a consequence of speed reduction of 0.5m/s** |  | **p value** | **Reduction in GRF_MAX_ in m as a consequence of speed reduction of 0.5m/s** | **Reduction in GRF_MAX_ in % as a consequence of speed reduction of 0.5m/s** |  | **Reduction in GRF_MAX_ in m as a consequence of speed reduction of 0.5m/s** | **Reduction in GRF_MAX_ in % as a consequence of speed reduction of 0.5m/s** |
| Predictor *Gate_OFFSET_* [m] | <.0001 | 0.13 | 6 |  | <.0001 | 0.17 | 2 |  | -0.04 | 4 |
| Predictor *Gate_VERTICAL_* [m] | 0.67 |  |  |  | 0.46 |  |  |  |  |  |
|  |  |  |  |  |  |  |  |  |  |  |
| **Impulse [BWs]** | **p value** | **Reduction in Impulse in m as a consequence of speed reduction of 0.5m/s** | **Reduction in Impulse in % as a consequence of speed reduction of 0.5m/s** |  | **p value** | **Reduction in Impulse in m as a consequence of speed reduction of 0.5m/s** | **Reduction in Impulse in % as a consequence of speed reduction of 0.5m/s** |  | **Reduction in Impulse in m as a consequence of speed reduction of 0.5m/s** | **Reduction in Impulse in % as a consequence of speed reduction of 0.5m/s** |
| Predictor *Gate_OFFSET_* [m] | <.0001 | 0.87 | 27 |  | <.0001 | 0.42 | 6 |  | 0.45 | 21 |
| Predictor *Gate_VERTICAL_* [m] | 0.01 | -0.07 | 2 |  | 0.88 |  |  |  |  |  |

**References**

1. Florenes, T. W., Bere, T., Nordsletten, L., Heir, S. & Bahr, R. Injuries among male and female World Cup alpine skiers. *Br. J. Sports Med.* **43**, 973–978 (2009).

2. Bere, T., Florenes, T. W., Nordsletten, L. & Bahr, R. Sex differences in the risk of injury in World Cup alpine skiers: a 6-year cohort study. *Br. J. Sports Med.* **48**, 36–49 (2013).

3. Haaland, B., Steenstrup, S. E., Bere, T., Bahr, R. & Nordsletten, L. Injury rate and injury patterns in FIS World Cup Alpine skiing (2006-2015): have the new ski regulations made an impact? *Br. J. Sports Med.* **50**, 32–36 (2015).

4. Alhammoud, M., Racinais, S., Rousseaux-Blanchi, M. P. & Bouscaren, N. Recording injuries only during winter competitive season underestimates injury incidence in elite alpine skiers. *Scand. J. Med. Sci. Sport.* **30**, 1177–1187 (2020).

5. Fröhlich, S. *et al.* Injury risks among elite competitive alpine skiers are underestimated if not registered prospectively, over the entire season and regardless of whether requiring medical attention. *Knee Surgery, Sport. Traumatol. Arthrosc.* (2020) doi:10.1007/s00167-020-06110-5.

6. Engebretsen, L. *et al.* Sports injuries and illnesses during the Winter Olympic Games 2010. *Br J Sport. Med* **44**, 772–780 (2010).

7. Soligard, T. *et al.* Sports injury and illness incidence in the PyeongChang 2018 Olympic Winter Games: A prospective study of 2914 athletes from 92 countries. *Br. J. Sports Med.* **53**, 1085–1092 (2019).

8. Soligard, T. *et al.* Sports injuries and illnesses in the Sochi 2014 Olympic Winter Games. *Br J Sport. Med* **49**, 441–447 (2015).

9. Gilgien, M., Crivelli, P., Spörri, J., Kröll, J. & Müller, E. Characterization of course and terrain and their effect on skier speed in World Cup alpine ski racing. *PLoS One* **10**, e0118119 (2015).

10. Gilgien, M., Crivelli, P., Spörri, J., Kröll, J. & Müller, E. Correction: Characterization of course and terrain and their effect on skier speed in World Cup alpine ski racing. *PLoS One* **10**, e0118119 (2015).

11. Yamazaki, J. *et al.* Analysis of a severe head injury in World Cup alpine skiing. *Med. Sci. Sport. Exerc.* **47**, 1113–1118 (2015).

12. Bere, T. *et al.* A systematic video analysis of 69 injury cases in World Cup alpine skiing. *Scand. J. Med. Sci. Sports* **24**, 667–677 (2013).

13. Petrone, N., Pollazzon, C. & Morandin, T. Structural Behaviour of Ski Safety Barriers during Impacts of an Instrumented Dummy. in *The Engineering of Sport 7* 633–642 (Springer Paris, 2008).

14. Gilgien, M., Spörri, J., Kröll, J., Crivelli, P. & Müller, E. Mechanics of turning and jumping and skier speed are associated with injury risk in men’s World Cup alpine skiing: a comparison between the competition disciplines. *Br. J. Sports Med.* **48**, 742–747 (2014).

15. Gilgien, M., Spörri, J., Kröll, J. & Müller, E. Effect of ski geometry and standing height on kinetic energy: Equipment designed to reduce risk of severe traumatic injuries in alpine downhill ski racing. *Br. J. Sports Med.* **50**, 8–13 (2016).

16. Kröll, J., Spörri, J., Gilgien, M., Schwameder, H. & Müller, E. Sidecut radius and the mechanics of turning: equipment designed to reduce risk of severe traumatic knee injuries in alpine giant slalom ski racing. *Br. J. Sports Med.* **50**, 14–19 (2016).

17. Spörri, J., Kröll, J., Gilgien, M. & Müller, E. Sidecut radius and the mechanics of turning-equipment designed to reduce risk of severe traumatic knee injuries in alpine giant slalom ski racing. *Br. J. Sports Med.* **50**, 14–19 (2016).

18. Gilgien, M. *et al.* Preventing injuries in alpine skiing giant slalom by shortening the vertical distance between the gates rather than increasing the horizontal gate offset to control speed. *Br. J. Sports Med.* **54**, 1042–1046 (2020).

19. Spörri, J., Kröll, J., Fasel, B., Aminian, K. & Müller, E. Course setting as a prevention measure for overuse injuries of the back in alpine ski racing: a kinematic and kinetic study of giant slalom and slalom. *Orthop. J. Sport. Med.* **4**, (2016).

20. Spörri, J., Kröll, J., Schwameder, H., Schiefermüller, C. & Müller, E. Course setting and selected biomechanical variables related to injury risk in alpine ski racing: an explorative case study. *Br. J. Sports Med.* **46**, 1072–1077 (2012).

21. Reid, R. C. A kinematic and kinetic study of alpine skiing technique in slalom. (Norwegian School of Sport Sciences, Oslo, Norway, 2010).

22. Gilgien, M., Spörri, J., Limpach, P., Geiger, A. & Müller, E. The Effect of Different Global Navigation Satellite System Methods on Positioning Accuracy in Elite Alpine Skiing. *Sensors (Basel)* **14**, 18433–18453 (2014).

23. Gilgien, M. *et al.* Determination of the centre of mass kinematics in alpine skiing using differential global navigation satellite systems. *J. Sports Sci.* **33**, 960–969 (2015).

24. Gilgien, M., Spörri, J., Chardonnens, J., Kröll, J. & Müller, E. Determination of external forces in alpine skiing using a differential global navigation satellite system. *Sensors (Basel)* **13**, 9821–9835 (2013).

25. Gilgien, M., Kröll, J., Spörri, J., Crivelli, P. & Müller, E. Application of dGNSS in alpine ski racing: Basis for evaluating physical demands and safety. *Front. Physiol.* **9**, 145 (2018).

26. Reid, R. C., Haugen, P., Gilgien, M., Kipp, R. W. & Smith, G. A. Alpine Ski Motion Characteristics in Slalom. *Front. Sport. Act. Living* (2020) doi:10.3389/fspor.2020.00025.

27. Supej, M., Kipp, R. & Holmberg, H. C. Mechanical parameters as predictors of performance in alpine World Cup slalom racing. *Scand. J. Med. Sci. Sports* **21**, e72-81 (2010).

28. Kröll, J., Spörri, J., Gilgien, M., Schwameder, H. & Müller, E. Effect of ski geometry on aggressive ski behaviour and visual aesthetics: equipment designed to reduce risk of severe traumatic knee injuries in alpine giant slalom ski racing. *Br. J. Sports Med.* **50**, 20–25 (2016).

29. Spörri, J., Kröll, J., Amesberger, G., Blake, O. & Müller, E. Perceived key injury risk factors in World Cup alpine ski racing an explorative qualitative study with expert stakeholders. *Br. J. Sports Med.* **46**, 1059–1064 (2012).

30. Elfmark, O. & Bardal, L. M. An Empirical Model of Aerodynamic Drag in Alpine Skiing. *Proceedings* **2**, 310 (2018).

31. Supej, M. *et al.* Aerodynamic drag is not the major determinant of performance during giant slalom skiing at the elite level. *Scand. J. Med. Sci. Sport.* (2013) doi:10.1111/sms.12007.

32. Wolfsperger, F., Rhyner, H. U. & Schneebeli, M. *Slope preparation and grooming. A handbook for practitioners*. (WSL Institute for Snow and Avalanche Research SLF, 2019).

33. Gilgien, M., Reid, R., Raschner, C., Supej, M. & Holmberg, H.-C. The Training of Olympic Alpine Ski Racers. *Front. Physiol.* **9**, 1772 (2018).
